# Comparison of electrospray and UniSpray, a novel atmospheric pressure ionization interface, for LC-MS/MS analysis of 81 pesticide residues in food and water matrices

**DOI:** 10.1007/s00216-019-01886-z

**Published:** 2019-05-31

**Authors:** Joseph Hubert Yamdeu Galani, Michael Houbraken, Marijn Van Hulle, Pieter Spanoghe

**Affiliations:** 1 School of Food Science and Nutrition, University of Leeds, Leeds, LS2 9JT UK; 2 Department of Plants and Crops, Faculty of Bioscience Engineering, Ghent University, Coupure Links 653, 9000 Ghent, Belgium; 3 Department of Agriculture and Veterinary Medicine, Université des Montagnes, P.O. Box 208, Bangangté, Cameroon; 4 Waters NV/SA, ConneXion Business Park, Brusselsesteenweg 500, 1731 Zellik, Belgium

**Keywords:** Pesticide residues, Electrospray, UniSpray, Mass spectrometry, Matrix effects, Process efficiency

## Abstract

In mass spectrometry, the type and design of ionization source play a key role on the performance of a given instrument. Therefore, it is of paramount importance to evaluate newly developed sources for their suitability to analyze food contaminants like pesticide residues. Here, we carried out a head-to-head comparison of key extraction and analytical performance parameters of an electrospray ionization (ESI) source with a new atmospheric pressure ionization source, UniSpray (US). The two interfaces were evaluated in three matrices of different properties (coffee, apple, and water) to determine if multiresidue analysis of 81 pesticides by QuEChERS extraction and LC-MS/MS analysis could be improved. Depending on the matrix and irrespective of the chemical class, US provided a tremendous gain in signal intensity (22- to 32-fold in peak area, 6- to 7-fold in peak height), a threefold to fourfold increase in signal-to-noise ratio, a mild gain in the range of compounds that can be quantified, and up to twofold improvement of recovery. UniSpray offered comparable linearity and precision of the analyses with ESI, and did not affect the ion ratio. A gain in sensitivity of many compounds was observed with US, but in general, the two ionization interfaces did not show significant difference in LOD and LOQ. UniSpray suffered less signal suppression; the matrix effect was in average 3 to 4 times more pronounced, but showed better values than ESI. With no effect on recovery efficiency, US improved the overall process efficiency 3 to 4 times more than ESI.

**Electronic supplementary material:**

The online version of this article (10.1007/s00216-019-01886-z) contains supplementary material, which is available to authorized users.

**Figure:**
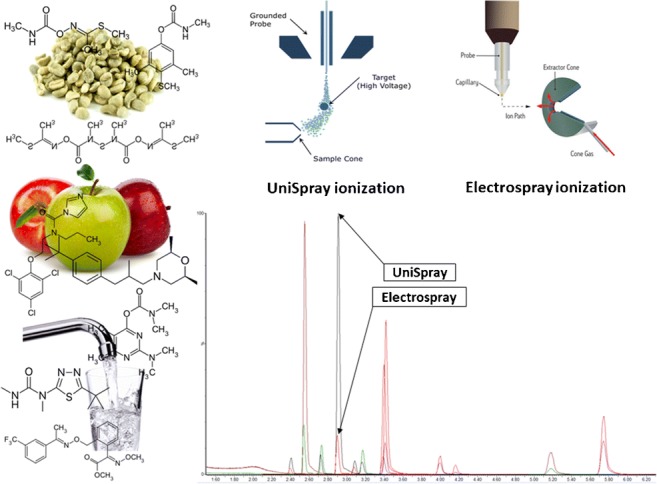
Graphical abstract

## Introduction

To gather surveillance data from the occurrence and background levels of both recognized and newly identified contaminants in foods, low limits of quantification (LOQs) are required, in order to estimate human daily intake for risk assessment [1]. Therefore, to analyze compounds like pesticide residues in foods and beverages, there is a constant need for more precise and accurate methods and instruments. The ability to quantitatively determine trace levels of residues in samples is essential to monitor and preserve consumer’s health in a precise and more effective way. Among the various techniques of analysis of pesticide residues in water and food items, liquid chromatography (LC) coupled by an atmospheric pressure ionization (API) source to tandem mass spectrometric (MS/MS) detection is the technique of choice, because it offers high throughput, selectivity, and sensitivity as well as its suitability for a wide range of compounds in various sample matrices [2–4]. It has been observed that the type and the design of an ionization source can have a significant influence on the performances of a bioanalytical method like LC-MS/MS [5]. Furthermore, several studies have demonstrated the differences on the ionization of specific classes of compounds and differences effects of the matrix, observed between different sources [6–11]. It is therefore of high interest for LC-MS/MS pesticide residue analysis, to evaluate the performances of newly introduced ionization sources in order to highlight their benefits and limitations in comparison with the source that is most commonly applied, i.e., electrospray ionization (ESI) [12].

UniSpray (US) ionization or impactor ionization is a novel atmospheric ionization technique developed by Waters Corporation that makes use of a high-velocity spray, created from a grounded nebulizer impacting on a high-voltage target (stainless steel rod), to ionize analytes in a similar fashion to ESI but promotes extra droplet break-up and desolvation via additional Coandă and vortex effects [13]. Comparatively with ESI, US was proven more performant in analysis of various compounds. The US interface showed a fivefold increase in method sensitivity, with an improved signal intensity, linearity, and repeatability on various matrices in comparison with ESI, for the analysis of prostaglandins and thromboxanes [14]. Similarly, for 24 pharmaceutical and biological compounds, US above ESI improved the dynamic range of analytes at lower concentrations and the sensitivity of late eluting compounds [12]. The novel source US generates very similar spectra compared with ESI, predominantly producing protonated or deprotonated species, but improves the intensity of the MS signal by more than twofold on average. The differences in source design between ESI and US have no significant effect on the adduct formation (e.g., proton, sodium, potassium adducts) and up-front fragmentation [6]. However, little is known on the performance of US for routine multiresidue analysis of pesticides in different matrices, as compared with the current largely used ESI.

Despite the numerous advantages of LC-API-MS/MS over other analytical techniques, the quantitative analysis of biological samples is complicated by the presence of matrix components that co-elute with the compound(s) of interest and can interfere with the ionization process in the mass spectrometer, causing ionization suppression or enhancement [15]. This phenomenon, called matrix effect (ME), was first described in 1993 [16] and until today, its mechanism is not fully understood. The ME is defined as the change in the signal intensity of an analyte in a matrix solution compared with the signal intensity in the corresponding solvent [17]. Matrix effects cause a compound’s response to differ when analyzed in a biological matrix, with signal suppression or enhancement effects, and therefore, must be determined and quantified to ensure acceptable quantitative results in pesticide residue analysis. The extent of ME can be influenced by some instrumental parameters such as the ionization source [18] and ionization mode [7]. Differences were observed in ME percentages of US and ESI analysis of pharmaceutical and biological compounds from plasma and bile [12]. These unpredictable effects are a regular problem for API sources [15], so the ME of novel sources must be investigated for analysis of specific compounds like pesticides in various matrices. Besides, the ME is used to describe the analyte ionization efficiency, while the efficiency of separating analyte from the sample is measured by the recovery. The process efficiency (PE) then summarizes the efficiency of sample preparation (extraction recovery) and analyte ionization during LC-MS/MS analysis (ME). Hence, PE is the suitable parameter for assessing the overall performance of an analysis method [2].

Therefore, this study aimed at determining whether multiresidue analysis of pesticides in food and water on the same LC-MS/MS system can be improved with US, comparatively with the commonly used ESI. The selected active ingredients (a.i.) belong to largely used pesticide classes, i.e., insecticides, fungicides, herbicides, nematicides, and acaricides, and are a good representative selection for such study because of their variable hydrophobic character and their different physicochemical properties. Matrices with different analytical challenges, textures, and physicochemical properties, and also largely consumed, including an agricultural dry product (coffee), a fresh product (apple), and water, were selected. Key extraction and analytical performance parameters like signal intensity, signal-to-noise (S/N) ratio, linearity, accuracy, precision, relative abundance (ion ratio), range of a.i., extraction recovery, sensitivity, and ME, as well as process performance parameters like recovery efficiency (RE) and PE were evaluated and compared.

## Materials and methods

### Reagents

Analytical grade reagents of above 99% purity were used in the experiments. UPLC-grade acetonitrile was procured from VWR Chemicals (Leuven, Belgium), and anhydrous magnesium sulfate, disodium hydrogen sesquihydrate, trisodium citrate dehydrate, sodium chloride, and pesticide a.i. standards were purchased from Sigma-Aldrich (Bornem, Belgium). The 15-ml-d-SPE tubes as well as Sep-Pak cartridge C18 column were obtained from Waters (Milford, MA, USA). Water was produced locally though a Milli-Q purification system.

### Sample collection and preparation

Raw coffee beans and apples were purchased in organic shops in Ghent, Belgium. Traces of epoxiconazole, imidacloprid, pyraclostrobine, thiametoxam, and hexythiazox were found in blank coffee samples, as well as pyrimethanil in blank coffee and apple samples. They were used for correction of corresponding signals obtained in spiked samples. Extraction and clean-up were performed using the QuEChERS method commonly used in the multiresidue analysis of food matrices. Approximately 50 g of sample was ground to powder or paste using a household mill equipped with a stainless steel knife (Krups, Fleurus, Belgium). Precisely 2 g of coffee powder or 10 g of apple paste was weighed into a 50-ml Teflon-capped centrifuge tube, 8 ml of Milli-Q water was added in the coffee powder, and then 15 ml of acetonitrile was added to each sample, and the mixture was vigorously shaken for 1 min. A mixture of disodium hydrogen citrate sesquihydrate (0.75 g), trisodium citrate dihydrate (1.5 g), sodium chloride (1.5 g), and anhydrous magnesium sulfate (6 g) was added to the extract into the tube, which was agitated for 3 min at 300 rpm on a shaker (Edmund Bühler, Hechingen, Germany). The tube was centrifuged for 5 min at 10,000 rpm (Eppendorf, Leipzig, Germany) and the supernatant was collected. For clean-up of the coffee extract, 7 ml of the supernatant was pipetted into a 15-ml-d-SPE tube packed with primary secondary amines (PSA) and octadecyl (C18). The content of the tube was then shaken for 1 min, centrifuged for 5 min at 3000 rpm, and the supernatant collected. For LC-MS/MS analysis, 1 ml of the supernatant was diluted 10 times with Milli-Q water, and 2 ml of the diluted solution was sampled into a screw cap autosampler vial for chromatography analysis. For the other sample sets (pre-extraction spiked samples), before the step of addition of 15 ml of acetonitrile, samples were spiked at 0.01 mg/l with each pesticide standard. The spiked samples were left for 1 h at room temperature to allow pesticide absorption into sample before being subjected to the extraction, clean-up process, and analysis as described previously.

For water samples, Sep-Pak cartridges were used for extracting the pesticides spiked in Milli-Q water [19]. Methanol (1 ml) and water (1 ml) were consecutively used to activate the cartridge before loading the sample. One liter of Milli-Q water sample was passed through the cartridge and pesticides were retained on the column. The pesticides were then desorbed with 10 mL of acetonitrile; the extract was diluted 10 times with Milli-Q water and sampled for chromatography analysis. The other water sample sets (pre-extraction spiked samples) were spiked at 0.01 mg/l with each pesticide standard before Sep-Pak cartridge extraction as described previously.

### Liquid chromatography tandem mass spectrometry analysis

The protocol from Galani et al. [20] was followed. The equipment consisted of a Waters Acquity UPLC module coupled to a Waters Xevo TQD tandem triple quadrupole mass spectrometer, equipped with ESI or US ion source (Waters, Milford, MA, USA). Separation was carried out through a HSS T3 column (100 mm × 2.1 mm, 1.8 μm) (Waters) maintained at 40 °C. The injection volume was 10 μl; mobile phase A consisted of a 0.1% formic acid solution in water while mobile phase B was acetonitrile with 0.1% formic acid. The flow rate was set at 0.4 ml/min with a run time of 10 min. The separation started with an initial gradient of 98% mobile phase A for 0.25 min, followed by a linear gradient to 98% mobile phase B from 0.25 to 7 min which was maintained for 1 min. Then, a linear gradient was used to 98% mobile phase A and column was reconditioned for 1 min. The analyses were performed with US and ESI consecutively with less than 24-h interval gap between the two interfaces, with the parameters presented in Table 1. The ESI capillary position in relation to the mass spectrometer aperture as well as the US source protrusion of the capillary within the nebulizer tube and the vertical and horizontal position of the probe tip towards the metal rod were optimized for achieving best results. Analyses of pesticides were performed in positive ion mode, except for fludioxonil and 2,4-D, which were analyzed in negative ion mode. The analytes were monitored and quantified using multiple reaction monitoring (MRM). The optimization of the MS/MS conditions, identification of the precursor and product ions, and selection of the cone and collision voltages were performed with direct infusion of their individual standard solutions prepared at 1 mg/ml in acetonitrile/water (10/90). After the optimization of the collision cell energy, two different m/z transitions were selected for each analyte, one for quantification (QIT) and one for confirmation (CIT). The dwell time was calculated automatically. Parameters of acquisition method are summarized in Table 2. MassLynx 4.1 software (Waters, Milford, MA, USA) was used for the LC-MS/MS system control and data acquisition and analysis.

**Table 1:** Parameters of the UniSpray and electrospray ionization sources

Source	UniSpray	Electrospray
Source temperature (°C)	150	150
Desolvation temperature (°C)	600	600
US rod voltage/ESI capillary voltage (kV)	± 3	± 2
Cone gas flow (l/h)	20	50
Desolvation gas flow (l/h)	1000	1000

**Table 2:** Parameters of acquisition method of LC-MS/MS analysis of 81 pesticide active ingredients

Sr. no.	Analyte	Retention time (min)	Precursor ion (m/z)	Cone voltage (eV)	Ionization mode	Dwell time (s)	Product ion 1 (m/z)	Collision energy 1 (eV)	Product ion 2 (m/z)	Collision energy 2 (eV)
1	Methomyl	2.40	163	20	+	0.017	88*	10	106	10
2	Methiocarb	4.00	226	22	+	0.015	121	22	169*	10
3	Fenpropimorph	3.44	304.2	50	+	0.015	57.2	30	147.2*	28
4	Tebuthiuron	2.90	229	30	+	0.015	116	16	172*	18
5	Pirimicarb	2.54	239.1	28	+	0.017	72	28	182.1*	15
6	Thiodicarb	3.17	355	20	+	0.015	87.9*	16	107.9	16
7	Prochloraz	4.17	376	16	+	0.015	70.1*	34	307.1	16
8	Trifloxystrobin	5.76	409	28	+	0.073	145	40	186*	16
9	Acetamiprid	2.71	223	34	+	0.015	56.1	15	126*	20
10	Thimetoxam	3.08	292	22	+	0.038	132	22	211.2*	12
11	Difenconazole	5.19	406	40	+	0.015	111.1	60	251.1*	25
12	Pyrimethanil	3.38	200	45	+	0.015	82	24	107*	24
13	Ametryn	3.10	228.1	32	+	0.013	68.1	36	186.1*	18
14	Boscalid	4.37	342.9	35	+	0.013	139.9*	20	307	20
15	Butachlor	6.11	312.2	20	+	0.067	57.3	22	238.2*	12
16	Carbaryl	3.36	202	22	+	0.08	117	28	145*	22
17	Dimethomorph	4.00	388.1	35	+	0.013	165	30	300.9*	20
18	Hexaconazole	4.67	314	16	+	0.013	70.1*	34	159	22
19	Malathion	4.53	331	20	+	0.013	99	24	127*	12
20	Propoxur	3.18	210	15	+	0.013	111*	16	168	10
21	Spinosad A	4.10	732.6	50	+	0.013	98.1	59	142*	31
22	Spinosad D	4.36	746.5	45	+	0.013	98.1	53	142*	31
23	Spiroxamine	3.45	298	32	+	0.013	100	32	144*	20
24	Thiabendazole	2.36	202	45	+	0.013	131	30	175*	25
25	Thifensulfuron-methyl	2.49	388	30	+	0.015	56	40	167*	15
26	Carbofuran	3.20	222.1	28	+	0.012	123*	16	165.1	16
27	Dimethoate	2.66	230.1	18	+	0.012	125	20	199*	10
28	Diuron	3.53	233	28	+	0.012	46.3	14	72.1*	18
29	Ethoprophos	4.29	243.2	26	+	0.012	97	31	131*	20
30	Fenamiphos	4.25	304.1	30	+	0.012	202.1	36	217.1*	24
31	Fenbuconazole	4.63	337	32	+	0.012	70.1*	20	125	36
32	Fludioxonil	4.14	246.8	50	–	0.013	126*	30	180	28
33	Metalaxyl	3.41	280.1	20	+	0.012	192.1	17	220.1*	13
34	Metsulfuron methyl	3.07	382	22	+	0.02	167*	16	198	22
35	Monocrotophos	2.39	224.1	20	+	0.163	98.1	12	127.1*	16
36	Pendimethalin	6.23	282.2	20	+	0.028	194	18	212.2*	10
37	Pyrazosulfuron-ethyl	4.03	415	22	+	0.012	82.9	45	182*	20
38	Triazophos	4.60	314.1	25	+	0.012	118.9	35	161.9*	18
39	Azoxystrobin	4.19	404	22	+	0.015	329	30	372*	15
40	Bentazon	3.29	241.4	21	+	0.015	107.2	26	199.1*	12
41	Bitertanol	4.69	338.1	15	+	0.015	70.1*	8	99.1	16
42	Cadusafos	5.15	271.1	22	+	0.015	131	22	159*	16
43	Chlorotoluron	6.26	213	20	+	0.03	72*	20	140	30
44	Cymoxanil	2.77	199	17	+	0.015	111	18	128*	8
45	Iprodione	4.63	330	15	+	0.015	244.7*	16	288	15
46	Linuron	4.06	249.1	31	+	0.015	159.9*	18	181.8	16
47	Oxamyl	2.35	237	15	+	0.163	72*	10	90	10
48	Propanil	3.89	217.9	34	+	0.015	127	22	161.9*	16
49	Tebuconazole	4.51	308	40	+	0.015	70.1*	22	125	40
50	Terbutryn	3.48	242.1	34	+	0.015	91	28	186.1*	20
51	Tiofanate-methyl	3.10	343	22	+	0.015	93	46	151*	22
52	Kresoxim-methyl	4.97	314.1	18	+	0.017	116	12	206*	7
53	Carbendazim	2.27	192.1	27	+	0.08	132.1	28	16.1*	18
54	Diazinon	5.16	305	31	+	0.017	96	35	169*	22
55	Imidacloprid	2.63	256.1	34	+	0.038	175.1*	20	209.1	15
56	Imazalil	2.99	297	34	+	0.02	69*	22	159	22
57	Metribuzin	3.12	215	35	+	0.012	89	20	131*	18
58	Profenofos	5.63	372.9	36	+	0.017	127.9	40	302.6*	20
59	Propiconazole	4.78	342	40	+	0.017	69	22	159*	34
60	Pyrachlostrobin	5.32	388.1	25	+	0.017	163	25	193.9*	12
61	Triadimenol	3.94	296.1	15	+	0.017	70.2*	10	99.1	15
62	Terbufos	6.07	289	12	+	0.017	57.2	22	103*	8
63	Thiacloprid	2.89	253	25	+	0.071	90.1	40	126*	20
64	Penconazole	4.66	284	28	+	0.052	70.1*	16	159	34
65	Pirimiphos-methyl	5.13	306.1	30	+	0.052	108.1*	32	164.1	22
66	Tebufenozide	4.94	353.1	13	+	0.052	133	20	297.1*	8
67	Spirodiclofen	6.98	411.1	25	+	0.108	71.2*	13	313	13
68	Cyflufenamid	5.71	413.2	30	+	0.052	203	35	295.1*	15
69	Temephos	6.35	466.8	32	+	0.052	125*	38	418.9	22
70	2,4-D	3.52	160.7	50	−	0.071	88.9	20	124.9*	18
71	Chlorpyrifos	3.38	349.9	30	+	0.037	97*	32	198	20
72	Cyanazine	3.12	241.1	35	+	0.03	96	25	214	17
73	Terbutylazine	4.10	230	28	+	0.03	96	28	174*	16
74	Propazine	3.97	230.2	34	+	0.03	146.1	24	188.1*	18
75	Atrazine	2.48	174	30	+	0.038	96*	20	103.9	20
76	Simazine	3.10	202	34	+	0.03	96	22	124*	16
77	Isoproturon	3.52	207.3	34	+	0.03	46	16	72*	16
78	Fenoxycarb	4.74	302.1	22	+	0.03	88	20	116.1*	11
79	Epoxiconazole	4.34	330	28	+	0.03	101	50	121*	22
80	Benalaxyl	4.97	326.1	20	+	0.064	91	34	148*	20
81	Hexythiazox	6.31	353	24	+	0.136	168.1	26	228.1*	14

*Transition used for quantification (QIT)

### Evaluation of the performance

Eight replicate injections of each sample were performed. To determine the linearity, five different concentrations of the stock solution (0.1, 0.05, 0.01, 0.005, 0.001 mg/l) were prepared by dilution with acetonitrile/water (10/90) to form a calibration curve. The signal intensity (peak area and peak height), S/N ratio, and relative abundance (ion ratio) of the QIT were calculated by the software. The sensitivity was evaluated by determining the limit of detection (LOD) and the limit of quantification (LOQ), which were statistically calculated based on the t_99_S_LLMV_ method [21], by multiplying the standard deviation of the detected pesticide concentration at 0.01 mg/l from the eight replicates by 2.998 (for LOD) and 10 (for LOQ). The accuracy (percentage extraction recovery, %recovery) was calculated by dividing the recovered concentrations by spiked concentration. Finally, the precision (percentage relative standard deviation, %RSD) was obtained by dividing the standard deviation by the average calculated concentration.

Matrix effect was determined by post-extraction spike matrix comparison [2]. A set of blank samples was spiked after the procedure of pesticide extraction, at 0.01 mg/l and thoroughly mixed. These post-extraction spiked samples were then diluted 10 times and analyzed as previously described. The peak area of the pesticide in solvent (A), the peak area of the pesticide in post-extraction spiked samples (B), and the peak area of the pesticide in pre-extraction spiked samples (C) were used to calculate the matrix effect (ME), recovery efficiency (RE), and process efficiency (PE) as follows [22]:

$$ \mathrm{ME}\ \left(\%\right)=\mathrm{B}/\mathrm{A}\times 100 $$

$$ \mathrm{RE}\ \left(\%\right)=\mathrm{C}/\mathrm{B}\times 100 $$

$$ \mathrm{PE}\ \left(\%\right)=\mathrm{C}/\mathrm{A}\times 100=\left(\mathrm{ME}\times \mathrm{RE}\right)/100 $$

A value of 100% indicates that there is no absolute ME; if the value is above 100%, there is a signal enhancement and there is signal suppression if the value is < 100%.

### Statistical analysis

The number of times (fold) US was higher or lower than ESI value was obtained by dividing each US value by its counterpart ESI value. To determine statistically if the US improved the performance of analyses, the means of different parameters were compared between US and ESI using a one-tailed paired Student’s *t* test; *p* values less than 0.05, 0.01, and 0.001 were considered significant, highly significant, and very highly significant, respectively. The software SPSS Statistics 19.0 (IBM Corporation, NY, USA) was used.

## Results and discussion

### Linearity

For the tested concentration range (0.001 to 0.1 mg/l), a very highly significant difference (*p* = 0.000005) was observed between the values of US and ESI (Electronic Supplementary Material, ESM) but in both cases, the *r*^2^ values were very good: they ranged from 0.9976 to 0.9999 with US, and from 0.9983 to 0.9999 with ESI. The significant difference between ESI and US may result from the fact that the *r*^2^ values are very close to each other. Similar linearity with *r*^2^ values ranging from 0.994 to 0.999 but with no significant difference between US and ESI was previously reported for pharmaceutical compounds [14].

### Signal intensity

There was a very highly significant difference in peak areas obtained with the two interfaces in the three matrices (*p* = 0.0000002, 0.000035, and 0.000001 in apple, coffee, and water, respectively); US allowed a tremendous gain in intensity, up to 22.4 times in apple (spinosad D), 31.6 times in coffee (spinosad D), and 24.5 times in water (kresoxim-methyl). In average, the gain in peak area with US was 6.4-fold in apple, 7.0-fold in coffee, and 7.2-fold in water (Table 3). Similarly, a highly significant increase of peak height was obtained with US (*p* = 0.0000001, 0.000033, and 0.000002 in apple, coffee, and water, respectively), and peak 21.3 times higher was obtained with spinosad D in apple, 21.1 times higher with spiroxamine in coffee, and 20.3 times higher with kresoxim-methyl in water. In general, US allowed a peak height gain of 6.3-fold in apple, 6.8-fold in coffee, and 6.9-fold in water (see ESM). A general increase in peak area ranging from a factor 1.1 to 15 with an average around 2 was observed with US for analysis of prostaglandins and thromboxanes [14]. Likewise, US showed an intensity gain of a factor 2.2 compared with ESI when analyzing by infusion, a mix of 22 pharmaceutical compounds. The design of the UniSpray source helps to promote droplet break-up and desolvation which has a significant effect on signal intensity [6].

**Table 3:** Comparison of performance parameters between UniSpray and electrospray sources for analysis of 81 pesticide residues in apple, coffee, and water

		Peak area			Signal-to-noise ratio			Limit of quantification (mg/kg)			Matrix effect (%)			Process efficiency (%)		
Sr. no.	Analyte	US	ESI	Fold	US	ESI	Fold	US	ESI	Fold	US	ESI	Fold	US	ESI	Fold
Apple																
1	Methomyl	7385.8	1402.6	5.3	2641.3	634.6	4.2	0.0023	0.0037	1.6	38.1	5.6	6.8	40.9	5.2	7.8
2	Methiocarb	4954.9	2879.1	1.7	1661.4	1377.8	1.2	0.0027	0.0011	0.4	98.1	8.9	11.1	63.3	5.9	10.7
3	Fenpropimorf	63,342.1	5665.3	11.2	3375.3	707.8	4.8	0.0018	0.0019	1.1	634.1	45.4	14.0	59.3	4.8	12.2
4	Tebuthiuron	93,105.1	8790.3	10.6	9246.3	2155.1	4.3	0.0034	0.0025	0.7	113.7	8.9	12.7	77.3	5.8	13.3
5	Pirimicarb	46,063.6	4118.8	11.2	4816.9	708.5	6.8	0.0012	0.0014	1.2	82.7	8.5	9.7	53.4	5.2	10.2
6	Thiodicarb	10,020.5	3430.5	2.9	1749.6	1004.6	1.7	0.0032	0.0021	0.7	101.3	10.0	10.1	56.2	5.7	9.8
7	Prochloraz	4462.3	348.8	12.8	917.4	280.1	3.3	0.0024	0.0023	1.0	90.7	3.7	24.3	49.6	2.3	21.8
8	Trifloxystrobin	18,436.0	9741.4	1.9	3772.6	2109.1	1.8	0.0013	0.0017	1.3	58.9	5.9	10.0	37.3	5.1	7.3
9	Acetamiprid	11,007.4	4581.9	2.4	985.1	811.3	1.2	0.0021	0.0037	1.8	70.0	8.3	8.5	49.2	5.7	8.6
10	Thifensulfuron	5531.9	1962.8	2.8	1286.8	1067.0	1.2	0.0094	0.0079	0.8	111.8	8.2	13.6	92.5	5.7	16.3
11	Difenconazole	12,020.6	1625.0	7.4	991.5	340.5	2.9	0.0030	0.0023	0.8	100.2	5.3	19.0	67.4	4.2	16.0
12	Pyrimethanil	77,256.4	6833.3	11.3	2214.4	723.0	3.1	0.0049	0.0043	0.9	616.3	58.8	10.5	106.2	10.0	10.6
13	Ametryn	89,324.5	9614.6	9.3	6582.8	1482.8	4.4	0.0021	0.0012	0.5	9.6	7.8	1.2	6.0	5.5	1.1
14	Boscalid	6735.0	1496.3	4.5	1134.6	816.9	1.4	0.0049	0.0036	0.7	10.3	6.4	1.6	6.6	4.5	1.4
15	Butachlor	1494.8	594.9	2.5	519.0	289.5	1.8	0.0026	0.0025	1.0	9.0	4.8	1.9	6.3	4.3	1.5
16	Carbaryl	3237.5	830.8	3.9	775.5	245.4	3.2	0.0030	0.0036	1.2	9.1	7.9	1.2	5.4	5.1	1.1
17	Dimethomorph	7545.8	2771.1	2.7	878.3	348.4	2.5	0.0014	0.0027	2.0	11.2	8.9	1.3	5.7	4.9	1.2
18	Hexaconazole	13,795.1	1877.4	7.3	1342.1	665.3	2.0	0.0017	0.0023	1.3	9.4	6.5	1.5	6.0	5.1	1.2
19	Malathion	5152.8	2763.3	1.9	551.0	489.9	1.1	0.0023	0.0024	1.0	9.4	8.4	1.1	4.9	4.7	1.0
20	Propoxur	25,964.1	4711.5	5.5	2409.5	657.3	3.7	0.0018	0.0031	1.7	9.6	8.1	1.2	6.1	5.4	1.1
21	Spinosad A	19,849.1	1132.1	17.5	2821.5	255.0	11.1	0.0153	0.0043	0.3	497.3	19.6	25.4	596.8	16.1	41.0
22	Spinosad D	6631.9	296.0	22.4	5724.3	312.6	18.3	0.0267	0.0113	0.4	451.4	19.9	22.7	607.7	18.2	33.4
23	Spiroxamine	128,712.8	8507.8	15.1	3393.0	499.0	6.8	0.0015	0.0006	0.4	7.6	4.0	1.9	5.7	3.1	1.9
24	Thiabendazole	25,288.5	1441.9	17.5	2594.9	338.8	7.7	0.0005	0.0019	3.7	6.3	4.0	1.6	3.8	2.8	1.4
25	Thiametoxam	1474.5	626.4	2.4	274.5	169.3	1.6	0.0031	0.0029	0.9	5.1	5.1	1.0	4.4	4.2	1.1
26	Carbofuran	33,900.8	6401.8	5.3	2437.8	758.4	3.2	0.0027	0.0023	0.9	11.3	8.4	1.3	7.3	5.6	1.3
27	Dimethoate	11,479.1	2524.8	4.5	2561.3	888.4	2.9	0.0016	0.0019	1.2	8.8	7.4	1.2	6.0	4.8	1.2
28	Diuron	13,937.6	3965.1	3.5	1320.0	497.5	2.7	0.0040	0.0031	0.8	10.7	9.1	1.2	6.4	6.0	1.1
29	Ethoprophos	11,902.1	4205.5	2.8	654.9	525.3	1.2	0.0030	0.0019	0.6	12.2	9.0	1.4	7.4	5.9	1.2
30	Fenamiphos	13,241.5	4096.0	3.2	1613.0	691.9	2.3	0.0015	0.0036	2.4	12.4	9.8	1.3	6.2	5.4	1.1
31	Fenbuconazole	6146.9	1449.5	4.2	3508.0	402.3	8.7	0.0043	0.0075	1.7	9.7	7.3	1.3	6.0	5.6	1.1
32	Fludioxonil	1149.3	77.0	14.9	1188.9	87.4	13.6	0.0039	0.0070	1.8	11.9	7.0	1.7	7.4	5.3	1.4
33	Metalaxyl	46,920.8	10,792.3	4.3	1949.1	640.4	3.0	0.0022	0.0024	1.1	12.3	10.5	1.2	7.2	6.3	1.1
34	Metribuzin	3075.0	266.6	11.5	118.0	28.6	4.1	0.0041	0.0088	2.1	11.1	9.1	1.2	7.1	5.8	1.2
35	Monocrotophos	14,489.8	3131.6	4.6	1925.6	876.4	2.2	0.0010	0.0019	2.0	5.9	6.2	0.9	5.3	4.6	1.2
36	Pendimethalin	258.0	362.3	0.7	39.8	218.5	0.2	0.0017	0.0020	1.2	3.8	2.6	1.5	2.3	2.6	0.9
37	Pyrazosulfuron-ethyl	4733.5	2202.9	2.1	3337.4	448.4	7.4	0.0068	0.0019	0.3	9.1	7.5	1.2	6.6	5.7	1.2
38	Triazophos	32,214.9	9234.3	3.5	1840.5	1782.6	1.0	0.0021	0.0027	1.3	11.0	10.4	1.1	6.8	7.2	0.9
39	Azoxystrobin	35,276.0	10,195.0	3.5	5861.1	1174.6	5.0	0.0019	0.0017	0.9	17.6	13.5	1.3	6.9	5.6	1.2
40	Bentazon	129.6	36.6	3.5	12.3	6.6	1.8	0.0170	0.0050	0.3	7.3	6.1	1.2	4.8	3.9	1.2
41	Bitertanol	2940.1	317.4	9.3	480.1	153.6	3.1	0.0021	0.0071	3.4	8.3	6.9	1.2	5.1	4.9	1.0
42	Cadusafos	12,629.0	3006.6	4.2	1614.9	612.3	2.6	0.0013	0.0025	2.0	10.4	6.7	1.5	6.6	4.5	1.5
43	Chlorpyrifos	171.5	223.4	0.8	60.6	81.8	0.7	0.0046	0.0039	0.8	4.4	2.3	2.0	3.5	2.5	1.4
44	Cymoxanil	1184.0	516.4	2.3	263.3	474.5	0.6	0.0132	0.0060	0.5	13.1	8.7	1.5	8.4	5.3	1.6
45	Iprodione	146.9	34.4	4.3	15.1	28.8	0.5	0.0210	0.0193	0.9	15.4	7.7	2.0	8.9	5.0	1.8
46	Linuron	962.5	541.4	1.8	79.4	201.9	0.4	0.0094	0.0078	0.8	12.4	6.4	1.9	8.2	4.7	1.7
47	Oxamyl	20,751.5	2503.4	8.3	7032.9	1632.5	4.3	0.0016	0.0007	0.4	6.7	7.0	1.0	5.5	5.2	1.1
48	Propanil	890.8	301.0	3.0	65.1	42.3	1.5	0.0105	0.0099	0.9	10.0	7.0	1.4	6.7	4.7	1.4
49	Tebuconazole	18,561.5	2459.8	7.5	899.3	480.1	1.9	0.0036	0.0023	0.6	9.3	7.1	1.3	5.6	5.0	1.1
50	Terbuthryn	119,793.4	12,769.8	9.4	7864.5	1170.0	6.7	0.0010	0.0011	1.1	8.8	6.8	1.3	5.6	4.9	1.1
51	Thiofanate-methyl	1440.3	109.4	13.2	76.4	11.4	6.7	0.0031	0.0279	8.9	12.4	13.1	0.9	3.8	6.2	0.6
52	Kresoxim-methyl	909.5	42.9	21.2	833.3	61.5	13.5	0.0070	0.0059	0.8	7.5	7.0	1.1	4.4	5.3	0.8
53	Carbendazim	42,296.1	3703.0	11.4	4126.0	621.5	6.6	0.0013	0.0024	1.8	9.2	8.3	1.1	5.6	5.1	1.1
54	Diazinon	87,076.9	8830.0	9.9	2348.4	1815.5	1.3	0.0010	0.0013	1.3	9.7	8.1	1.2	5.7	5.2	1.1
55	Imidacloprid	552.8	197.1	2.8	195.6	550.0	0.4	0.0040	0.0058	1.4	6.9	5.3	1.3	4.4	3.6	1.2
56	Imazalil	8718.3	612.1	14.2	303.1	60.4	5.0	0.0002	0.0004	1.7	0.9	0.4	2.2	0.6	0.3	1.7
57	Metsulfuron-methyl	4183.1	1813.9	2.3	546.5	617.5	0.9	0.0002	0.0003	1.3	1.0	0.9	1.1	0.5	0.5	1.0
58	Profenofos	671.3	452.6	1.5	203.0	179.3	1.1	0.0040	0.0022	0.6	4.9	2.2	2.2	3.1	1.7	1.8
59	Propiconazole	6274.9	914.9	6.9	123.6	46.5	2.7	0.0011	0.0040	3.6	8.9	6.6	1.3	5.5	4.6	1.2
60	Pyraclostrobine	26,157.5	6396.9	4.1	2072.5	728.0	2.8	0.0008	0.0012	1.6	7.6	3.9	1.9	4.8	3.4	1.4
61	Triadimenol	NQ	NQ	NQ	NQ	NQ	NQ	NQ	NQ	NQ	NQ	NQ	NQ	NQ	NQ	NQ
62	Terbufos	105.9	23.0	4.6	14.1	5.6	2.5	0.0050	0.0074	1.5	5.0	5.4	0.9	2.9	3.6	0.8
63	Thiacloprid	8151.4	5324.6	1.5	1063.1	1119.5	0.9	0.0034	0.0008	0.2	9.0	6.5	1.4	5.8	4.2	1.4
64	Penconazole	17,579.1	2300.1	7.6	2511.4	543.9	4.6	0.0013	0.0022	1.7	9.2	7.9	1.2	5.9	4.9	1.2
65	Pirimiphos-methyl	26,338.0	4964.0	5.3	3442.6	1079.0	3.2	0.0018	0.0016	0.9	9.6	6.3	1.5	6.0	3.8	1.6
66	Tebufenozide	3124.3	775.6	4.0	1616.4	665.8	2.4	0.0068	0.0067	1.0	10.9	10.1	1.1	7.3	6.4	1.1
67	Spirodiclofen	697.5	192.6	3.6	122.6	60.4	2.0	0.0333	0.0034	0.1	26.3	3.5	7.6	15.9	2.0	8.1
68	Cyflufenamid	1382.1	1102.3	1.3	345.9	318.8	1.1	0.0037	0.0022	0.6	12.6	6.6	1.9	7.8	4.0	2.0
69	Temephos	272.6	NQ	NQ	41.1	NQ	NQ	0.0118	NQ	NQ	14.1	NQ	NQ	9.2	NQ	NQ
70	2,4-D	10.9	6.0	1.8	27.9	23.3	1.2	0.0176	0.0078	0.4	5.1	3.5	1.5	7.6	5.8	1.3
71	Chlorotoluron	12,044.6	4175.9	2.9	1304.6	2212.6	0.6	0.0013	0.0011	0.8	9.5	7.4	1.3	6.3	4.8	1.3
72	Cyanazine	13,686.8	1460.0	9.4	2430.6	1154.8	2.1	0.0022	0.0021	0.9	8.2	8.0	1.0	5.5	5.0	1.1
73	Terbuthylazine	63.9	6.9	9.3	53.8	18.0	3.0	0.0180	0.0383	2.1	9.6	5.6	1.7	6.6	3.8	1.7
74	Propazine	10,320.6	2671.6	3.9	1420.5	1503.4	0.9	0.0019	0.0020	1.1	9.9	7.1	1.4	6.4	4.7	1.4
75	Atrazine	3605.0	205.8	17.5	413.6	69.6	5.9	0.0012	0.0032	2.6	6.7	5.8	1.2	4.3	3.9	1.1
76	Simazine	23,559.4	1596.6	14.8	1414.9	272.8	5.2	0.0018	0.0028	1.6	9.1	7.3	1.3	6.0	4.5	1.3
77	Isoproturon	28,866.4	7110.4	4.1	2945.3	1997.3	1.5	0.0010	0.0010	1.0	9.9	8.0	1.2	6.6	5.2	1.3
78	Fenoxycarb	4603.4	2528.9	1.8	1333.1	1551.9	0.9	0.0025	0.0022	0.9	10.1	6.9	1.5	6.7	4.3	1.6
79	Epoxiconazole	21,751.4	3142.0	6.9	1061.4	325.5	3.3	0.0022	0.0023	1.0	9.3	8.4	1.1	6.1	5.2	1.2
80	Benalaxyl	13,774.3	9182.6	1.5	1601.3	1670.8	1.0	0.0016	0.0008	0.5	10.5	7.9	1.3	6.9	5.0	1.4
81	Hexythiazox	1069.6	585.1	1.8	544.1	465.6	1.2	0.0018	0.0008	0.4	11.4	3.0	3.8	7.8	1.9	4.2
Minimum value		10.9	6.0	0.7	12.3	5.6	0.2	0.0002	0.0003	0.1	0.9	0.4	0.92	0.5	0.3	0.6
Maximum value		128,712.8	12,769.7	22.4	9246.3	2212.6	18.3	0.0333	0.0383	8.9	634.1	58.8	25.4	607.7	18.2	41.0
Average value		18,312.1	3039.0	6.4	1799.2	660.0	3.4	0.0047	0.0042	1.3	46.1	8.4	3.7	29.4	5.0	3.9
*p* value		0.0000002***			0.00000001***			0.283456			0.002046**			0.010766*		
Coffee																
1	Methomyl	9381.6	831.5	11.3	2432.4	499.5	4.9	0.0033	0.0032	1.0	70.8	4.1	17.1	52.0	3.1	16.7
2	Methiocarb	4193.4	1959.0	2.1	2306.5	652.9	3.5	0.0046	0.0020	0.4	90.2	6.7	13.5	53.6	4.0	13.4
3	Fenpropimorf	22,737.9	1371.1	16.6	1866.9	443.5	4.2	0.0008	0.0011	1.4	53.7	3.7	14.6	21.3	1.2	18.2
4	Tebuthiuron	77,355.5	6965.8	11.1	9033.4	1324.6	6.8	0.0033	0.0021	0.6	106.2	7.4	14.4	64.2	4.6	14.0
5	Pirimicarb	50,219.1	2940.8	17.1	5861.5	745.0	7.9	0.0021	0.0015	0.7	93.1	6.1	15.3	58.2	3.7	15.6
6	Thiodicarb	9075.0	3196.9	2.8	4095.9	1737.5	2.4	0.0030	0.0010	0.3	99.1	9.4	10.5	50.9	5.3	9.5
7	Prochloraz	4408.5	390.0	11.3	935.9	159.1	5.9	0.0027	0.0051	1.9	70.7	3.9	18.0	49.0	2.5	19.2
8	Trifloxystrobin	11,831.0	4101.3	2.9	3023.3	1363.5	2.2	0.0034	0.0006	0.2	31.2	2.7	11.5	24.0	2.1	11.2
9	Acetamiprid	10,674.0	3836.9	2.8	1092.8	1080.0	1.0	0.0026	0.0012	0.5	78.1	7.4	10.5	47.7	4.8	10.0
10	Thifensulfuron	218.8	NQ	NQ	39.6	NQ	NQ	0.0006	NQ	NQ	111.0	NQ	NQ	3.7	NQ	NQ
11	Difenconazole	8405.9	973.6	8.6	1044.0	197.8	5.3	0.0009	0.0017	1.9	62.9	3.6	17.3	47.1	2.5	18.6
12	Pyrimethanil	245,808.8	19,453.4	12.6	7454.3	872.5	8.5	0.0209	0.0096	0.5	312.2	26.4	11.8	337.8	28.4	11.9
13	Ametryn	93,374.5	7002.5	13.3	6577.9	1364.1	4.8	0.0009	0.0026	2.9	10.7	6.3	1.7	6.3	4.0	1.6
14	Boscalid	6047.1	1353.6	4.5	679.6	611.9	1.1	0.0035	0.0037	1.0	10.0	6.1	1.6	5.9	4.1	1.4
15	Butachlor	710.5	216.3	3.3	207.5	110.5	1.9	0.0020	0.0045	2.2	4.2	2.5	1.7	3.0	1.6	1.9
16	Carbaryl	3309.0	601.1	5.5	1351.4	656.0	2.1	0.0021	0.0051	2.4	9.5	5.7	1.7	5.5	3.7	1.5
17	Dimethomorph	7410.9	2175.4	3.4	808.0	395.0	2.0	0.0017	0.0030	1.8	11.6	7.5	1.5	5.6	3.8	1.5
18	Hexaconazole	12,384.3	1241.0	10.0	1442.4	225.5	6.4	0.0025	0.0011	0.4	8.9	5.8	1.6	5.4	3.4	1.6
19	Malathion	5409.6	2340.8	2.3	659.8	614.6	1.1	0.0043	0.0022	0.5	8.8	6.5	1.4	5.2	4.0	1.3
20	Propoxur	27,407.6	3629.1	7.6	2802.5	707.1	4.0	0.0021	0.0021	1.0	10.5	6.3	1.7	6.4	4.1	1.6
21	Spinosad A	878.3	65.0	13.5	581.0	72.9	8.0	0.0017	0.0043	2.5	8.4	1.7	4.8	2.7	0.5	4.9
22	Spinosad D	343.1	10.9	31.6	477.4	16.3	29.4	0.0048	0.0080	1.7	9.0	1.0	8.9	3.4	0.3	10.3
23	Spiroxamine	1156.4	57.9	20.0	44.5	36.6	1.2	0.0001	0.0001	0.7	2.7	1.2	2.2	0.1	0.0	2.5
24	Thiabendazole	8095.9	702.4	11.5	935.8	170.8	5.5	0.0004	0.0010	2.4	2.7	2.6	1.0	1.2	1.4	0.9
25	Thiametoxam	2719.6	734.0	3.7	500.6	276.8	1.8	0.0025	0.0024	1.0	11.7	6.4	1.8	8.2	4.9	1.7
26	Carbofuran	32,023.0	4953.0	6.5	2050.1	767.0	2.7	0.0022	0.0019	0.8	11.3	6.7	1.7	6.9	4.3	1.6
27	Dimethoate	12,521.8	2010.4	6.2	2511.9	1542.9	1.6	0.0020	0.0016	0.8	10.6	6.0	1.8	6.5	3.8	1.7
28	Diuron	14,390.9	3025.4	4.8	1602.8	396.1	4.0	0.0038	0.0018	0.5	11.1	7.2	1.5	6.6	4.6	1.4
29	Ethoprophos	13,182.4	3082.6	4.3	732.4	1122.6	0.7	0.0042	0.0020	0.5	13.3	7.1	1.9	8.2	4.4	1.9
30	Fenamiphos	11,437.1	2331.0	4.9	1761.9	700.3	2.5	0.0031	0.0016	0.5	12.8	7.3	1.8	5.3	3.1	1.7
31	Fenbuconazole	5270.8	921.1	5.7	2833.3	1060.3	2.7	0.0024	0.0033	1.4	8.6	5.7	1.5	5.2	3.6	1.4
32	Fludioxonil	836.3	NQ	NQ	897.0	NQ	NQ	0.0034	NQ	NQ	8.6	4.7	1.8	5.4	NQ	NQ
33	Metalaxyl	48,720.0	8535.3	5.7	3829.9	889.1	4.3	0.0016	0.0031	2.0	12.4	8.1	1.5	7.5	5.0	1.5
34	Metribuzin	2625.8	182.1	14.4	146.0	16.8	8.7	0.0046	0.0067	1.5	9.7	6.5	1.5	6.0	3.9	1.5
35	Monocrotophos	17,231.9	1960.9	8.8	3051.5	658.1	4.6	0.0017	0.0011	0.6	10.9	4.6	2.4	6.3	2.9	2.2
36	Pendimethalin	87.1	82.8	1.1	33.9	56.5	0.6	0.0012	0.0007	0.5	1.1	0.6	2.0	0.8	0.6	1.3
37	Pyrazosulfuron-ethyl	644.8	257.3	2.5	856.9	431.8	2.0	0.0020	0.0011	0.6	10.0	6.8	1.5	0.9	0.7	1.4
38	Triazophos	30,976.8	6338.3	4.9	2761.5	1174.0	2.4	0.0017	0.0022	1.4	10.4	7.7	1.4	6.5	4.9	1.3
39	Azoxystrobin	31,921.4	7957.6	4.0	5294.9	2101.5	2.5	0.0015	0.0017	1.1	11.1	7.5	1.5	6.3	4.4	1.4
40	Bentazon	75.3	NQ	NQ	6.0	NQ	NQ	0.0086	NQ	NQ	7.2	NQ	NQ	2.8	NQ	NQ
41	Bitertanol	2260.5	241.9	9.3	892.0	88.1	10.1	0.0020	0.0060	3.0	6.4	6.3	1.0	3.9	3.7	1.0
42	Cadusafos	10,881.8	2119.0	5.1	1096.3	501.5	2.2	0.0018	0.0013	0.7	9.7	5.4	1.8	5.7	3.2	1.8
43	Chlorpyrifos	61.6	47.5	1.3	18.0	22.8	0.8	0.0035	0.0010	0.3	1.4	0.5	2.6	1.2	0.5	2.4
44	Cymoxanil	801.9	221.8	3.6	129.6	486.1	0.3	0.0074	0.0036	0.5	12.0	5.3	2.2	5.7	2.3	2.5
45	Iprodione	77.9	14.6	5.3	7.4	21.3	0.3	0.0115	0.0081	0.7	10.7	3.5	3.0	4.7	2.1	2.2
46	Linuron	846.3	396.6	2.1	140.0	74.6	1.9	0.0114	0.0038	0.3	11.7	5.6	2.1	7.2	3.4	2.1
47	Oxamyl	5348.0	605.6	8.8	1745.0	169.0	10.3	0.0006	0.0007	1.2	2.4	2.0	1.2	1.4	1.3	1.1
48	Propanil	721.6	214.6	3.4	34.0	168.3	0.2	0.0136	0.0064	0.5	9.6	5.9	1.6	5.5	3.3	1.6
49	Tebuconazole	16,540.5	1918.3	8.6	1362.0	322.6	4.2	0.0027	0.0042	1.5	8.7	6.6	1.3	5.0	3.9	1.3
50	Terbuthryn	97,963.9	8753.1	11.2	6528.8	1541.9	4.2	0.0007	0.0007	1.1	8.1	5.8	1.4	4.6	3.4	1.4
51	Thiofanate-methyl	NQ	NQ	NQ	NQ	**NQ**	NQ	NQ	NQ	NQ	**NQ**	NQ	NQ	NQ	NQ	NQ
52	Kresoxim-methyl	772.5	NQ	NQ	492.8	NQ	NQ	0.0086	NQ	NQ	7.6	4.4	1.7	3.8	NQ	NQ
53	Carbendazim	49,837.9	3356.6	14.8	8293.3	1392.1	6.0	0.0022	0.0017	0.8	14.1	9.0	1.6	6.6	4.6	1.4
54	Diazinon	78,696.3	6764.3	11.6	3813.9	1021.8	3.7	0.0011	0.0011	1.0	8.2	6.1	1.3	5.2	4.0	1.3
55	Imidacloprid	1256.3	351.6	3.6	291.5	902.0	0.3	0.0057	0.0085	1.5	14.5	8.8	1.7	10.1	6.4	1.6
56	Imazalil	NQ	250.3	NQ	NQ	18.5	NQ	NQ	0.0001	NQ	0.5	0.3	NQ	NQ	0.1	NQ
57	Metsulfuron-methyl	289.6	132.4	2.2	43.4	173.9	0.2	0.0001	0.0001	1.0	1.0	0.9	1.1	0.0	0.0	0.9
58	Profenofos	283.5	145.4	2.0	131.1	128.3	1.0	0.0016	0.0010	0.6	2.2	0.9	2.3	1.3	0.6	2.3
59	Propiconazole	5817.4	688.4	8.5	256.6	27.0	9.5	0.0021	0.0014	0.6	8.9	5.5	1.6	5.1	3.5	1.5
60	Pyraclostrobine	20,258.9	3853.9	5.3	2989.9	681.0	4.4	0.0008	0.0006	0.8	4.9	2.3	2.1	3.7	2.1	1.8
61	Triadimenol	NQ	68.3	NQ	NQ	39.0	NQ	NQ	0.0008	NQ	NQ	5.2	NQ	NQ	0.4	NQ
62	Terbufos	NQ	NQ	NQ	NQ	NQ	NQ	NQ	NQ	NQ	NQ	NQ	NQ	NQ	NQ	NQ
63	Thiacloprid	7987.1	5333.4	1.5	678.9	1270.9	0.5	0.0019	0.0013	0.7	9.5	6.6	1.4	5.7	4.2	1.4
64	Penconazole	17,318.0	2069.1	8.4	2570.8	484.3	5.3	0.0015	0.0027	1.8	8.6	7.1	1.2	5.8	4.4	1.3
65	Pirimiphos-methyl	27,371.5	3898.0	7.0	3504.4	693.9	5.1	0.0008	0.0011	1.3	8.7	4.5	1.9	6.3	3.0	2.1
66	Tebufenozide	3091.8	705.0	4.4	703.9	662.9	1.1	0.0077	0.0063	0.8	10.2	9.4	1.1	7.2	5.8	1.2
67	Spirodiclofen	444.3	146.9	3.0	162.6	54.4	3.0	0.0117	0.0035	0.3	16.3	1.4	11.5	10.1	1.5	6.8
68	Cyflufenamid	1260.4	657.6	1.9	224.9	252.6	0.9	0.0043	0.0026	0.6	10.2	3.6	2.9	7.1	2.4	3.0
69	Temephos	284.1	147.5	1.9	45.9	116.5	0.4	0.0055	0.0043	0.8	12.0	1.6	7.3	9.6	1.9	5.2
70	2,4-D	NQ	NQ	NQ	NQ	NQ	NQ	NQ	NQ	NQ	NQ	NQ	NQ	NQ	NQ	NQ
71	Chlorotoluron	11,744.5	4432.0	2.6	1315.4	904.5	1.5	0.0018	0.0026	1.4	10.1	8.4	1.2	6.2	5.1	1.2
72	Cyanazine	12,296.1	1806.1	6.8	1835.1	697.1	2.6	0.0014	0.0031	2.3	8.6	9.9	0.9	4.9	6.2	0.8
73	Terbuthylazine	52.5	6.0	8.8	51.3	27.4	1.9	0.0142	0.0478	3.4	8.9	4.1	2.2	5.4	3.3	1.6
74	Propazine	10,182.6	2981.5	3.4	2460.9	896.9	2.7	0.0011	0.0039	3.5	10.0	9.1	1.1	6.3	5.2	1.2
75	Atrazine	2733.5	194.0	14.1	249.4	40.5	6.2	0.0012	0.0040	3.4	5.4	5.6	1.0	3.2	3.6	0.9
76	Simazine	22,475.9	1725.1	13.0	1885.0	459.4	4.1	0.0007	0.0022	3.0	9.1	7.9	1.2	5.7	4.9	1.2
77	Isoproturon	28,025.4	7245.8	3.9	3256.9	1778.0	1.8	0.0025	0.0018	0.7	10.4	7.8	1.3	6.4	5.3	1.2
78	Fenoxycarb	4294.1	1624.6	2.6	2617.5	460.3	5.7	0.0022	0.0025	1.2	8.9	5.3	1.7	6.3	2.7	2.3
79	Epoxiconazole	22,672.9	3536.3	6.4	1487.9	330.9	4.5	0.0009	0.0027	3.1	9.0	8.9	1.0	6.3	5.9	1.1
80	Benalaxyl	12,915.4	8580.8	1.5	1117.5	1033.8	1.1	0.0018	0.0011	0.6	9.3	7.6	1.2	6.5	4.7	1.4
81	Hexythiazox	829.4	187.1	4.4	460.6	369.9	1.2	0.0055	0.0010	0.2	8.7	2.4	3.7	6.1	0.6	10.1
Minimum value		52.5	6.0	1.1	6.0	16.3	0.2	0.0001	0.0001	0.2	0.5	0.3	0.8	0.0	0.0	0.8
Maximum value		245,808.8	19,453.3	31.6	9033.4	2101.5	29.4	0.0209	0.0478	3.5	312.2	26.4	17.9	337.8	28.4	19.2
Average value		17,423.6	2475.7	7.0	1809.5	588.7	3.8	0.0035	0.0033	1.2	22.8	5.6	3.8	15.1	3.6	3.9
*p* value		0.000035***			0.00000001***			0.489345			0.000365***			0.005053**		
Water																
1	Methomyl	NQ	147.4	NQ	NQ	185.4	NQ	NQ	0.0007	NQ	NQ	8.1	NQ	NQ	0.6	NQ
2	Methiocarb	7668.6	3697.0	2.1	1894.0	768.4	2.5	0.0016	0.0018	1.1	103.0	8.1	12.8	98.0	7.6	12.9
3	Fenpropimorf	90,251.0	6355.8	14.2	4145.4	901.3	4.6	0.0014	0.0011	0.8	89.1	6.2	14.4	84.5	5.4	15.5
4	Tebuthiuron	158,648.0	11,565.6	13.7	14,357.8	3745.1	3.8	0.0017	0.0035	2.1	138.4	8.4	16.5	131.8	7.6	17.3
5	Pirimicarb	83,389.4	6321.4	13.2	6075.1	1095.0	5.5	0.0018	0.0033	1.9	103.3	8.3	12.5	96.6	8.0	12.0
6	Thiodicarb	12,108.9	4552.9	2.7	1945.1	1394.3	1.4	0.0008	0.0038	4.7	79.0	8.4	9.4	67.9	7.6	8.9
7	Prochloraz	12,539.3	860.4	14.6	1458.0	325.5	4.5	0.0025	0.0019	0.8	146.5	6.3	23.4	139.5	5.6	24.8
8	Trifloxystrobine	27,110.8	14,508.9	1.9	5097.9	1948.9	2.6	0.0015	0.0013	0.9	64.5	8.7	7.4	54.9	7.6	7.2
9	Acetamiprid	9723.4	4072.3	2.4	760.9	936.5	0.8	0.0009	0.0025	2.9	77.9	8.9	8.8	43.5	5.1	8.6
10	Thifensulfuron	3673.8	1104.8	3.3	1101.8	1131.0	1.0	0.0016	0.0021	1.3	110.0	6.2	17.8	61.4	3.2	19.3
11	Difenconazole	26,450.9	2598.1	10.2	1562.0	289.6	5.4	0.0028	0.0012	0.4	156.5	8.1	19.3	148.2	6.8	22.0
12	Pyrimethanil	126,667.0	9740.3	13.0	3932.1	624.8	6.3	0.0067	0.0026	0.4	477.4	41.4	11.5	174.1	14.2	12.2
13	Ametryn	135,517.4	12,982.6	10.4	8574.0	1938.1	4.4	0.0015	0.0016	1.1	10.0	7.8	1.3	9.1	7.4	1.2
14	Boscalid	8977.6	1993.3	4.5	1239.9	1846.4	0.7	0.0035	0.0023	0.7	9.8	6.5	1.5	8.7	6.0	1.4
15	Butachlor	2411.1	881.4	2.7	558.0	209.5	2.7	0.0039	0.0039	1.0	11.4	7.1	1.6	10.1	6.3	1.6
16	Carbaryl	4367.4	984.1	4.4	1941.8	1802.8	1.1	0.0025	0.0032	1.3	9.0	7.3	1.2	7.3	6.0	1.2
17	Dimethomorph	10,645.1	3744.8	2.8	1170.0	529.1	2.2	0.0021	0.0024	1.1	11.1	8.6	1.3	8.0	6.6	1.2
18	Hexaconazole	19,375.0	2103.3	9.2	1988.0	642.5	3.1	0.0022	0.0014	0.6	10.2	7.4	1.4	8.5	5.7	1.5
19	Malathion	8435.0	3978.5	2.1	810.4	573.3	1.4	0.0018	0.0020	1.1	9.1	7.4	1.2	8.1	6.8	1.2
20	Propoxur	30,316.1	5095.0	6.0	3457.0	1043.3	3.3	0.0020	0.0014	0.7	9.5	7.6	1.3	7.1	5.8	1.2
21	Spinosad A	1988.8	131.4	15.1	1219.3	190.8	6.4	0.0012	0.0017	1.5	5.3	1.3	4.0	6.1	1.1	5.5
22	Spinosad D	629.5	37.0	17.0	714.3	73.1	9.8	0.0029	0.0043	1.5	5.6	1.0	5.7	6.3	1.1	5.5
23	Spiroxamine	132,853.1	6827.1	19.5	3508.0	384.1	9.1	0.0013	0.0005	0.4	4.8	2.5	1.9	5.9	2.4	2.4
24	Thiabendazole	37,733.9	2049.9	18.4	2945.3	426.4	6.9	0.0018	0.0014	0.8	9.2	6.6	1.4	5.7	4.0	1.4
25	Thiametoxam	359.0	131.9	2.7	65.5	60.6	1.1	0.0008	0.0003	0.3	9.3	7.0	1.3	1.1	0.9	1.2
26	Carbofuran	43,743.4	6717.3	6.5	3314.5	932.3	3.6	0.0021	0.0014	0.6	10.9	7.3	1.5	9.4	5.8	1.6
27	Dimethoate	2782.9	471.5	5.9	610.4	860.8	0.7	0.0004	0.0003	0.7	10.8	7.1	1.5	1.4	0.9	1.6
28	Diuron	20,123.6	4494.9	4.5	1007.4	440.3	2.3	0.0039	0.0019	0.5	10.4	7.7	1.3	9.2	6.8	1.4
29	Ethoprophos	17,230.8	4685.1	3.7	1419.1	531.3	2.7	0.0033	0.0027	0.8	11.2	7.6	1.5	10.7	6.6	1.6
30	Fenamiphos	21,733.9	6209.1	3.5	3486.4	657.9	5.3	0.0027	0.0026	1.0	11.0	9.3	1.2	10.1	8.2	1.2
31	Fenbuconazole	9781.3	1741.5	5.6	2400.5	509.8	4.7	0.0034	0.0043	1.3	10.4	8.3	1.3	9.6	6.8	1.4
32	Fludioxonil	1825.6	106.3	17.2	907.4	80.8	11.2	0.0044	0.0044	1.0	12.5	7.4	1.7	11.7	7.3	1.6
33	Metalaxyl	66,743.8	13,058.8	5.1	3516.1	1616.4	2.2	0.0014	0.0018	1.2	11.3	8.6	1.3	10.3	7.6	1.3
34	Metribuzin	2690.3	181.9	14.8	178.8	19.9	9.0	0.0026	0.0043	1.7	11.2	8.0	1.4	6.2	3.9	1.6
35	Monocrotophos	4944.0	932.3	5.3	918.1	408.5	2.2	0.0003	0.0003	1.0	9.8	7.9	1.2	1.8	1.4	1.3
36	Pendimethalin	984.1	821.0	1.2	78.3	66.8	1.2	0.0035	0.0018	0.5	10.7	7.9	1.4	8.6	5.9	1.5
37	Pyrazosulfuron-ethyl	6984.3	2835.4	2.5	2100.1	860.8	2.4	0.0036	0.0039	1.1	8.8	6.9	1.3	9.8	7.3	1.3
38	Triazophos	44,538.8	10,791.4	4.1	2922.9	1441.1	2.0	0.0023	0.0017	0.7	10.6	9.6	1.1	9.4	8.4	1.1
39	Azoxystrobin	48,719.4	12,878.8	3.8	5734.6	1387.5	4.1	0.0026	0.0015	0.6	11.1	8.1	1.4	9.5	7.1	1.3
40	Bentazon	NQ	NQ	NQ	NQ	NQ	NQ	NQ	NQ	NQ	NQ	NQ	NQ	NQ	NQ	NQ
41	Bitertanol	4531.5	460.1	9.8	694.4	170.0	4.1	0.0023	0.0067	2.9	9.2	8.2	1.1	7.8	7.1	1.1
42	Cadusafos	18,517.9	3857.3	4.8	3121.6	833.1	3.7	0.0014	0.0007	0.5	10.9	6.4	1.7	9.6	5.7	1.7
43	Chlorpyrifos	597.9	457.0	1.3	136.1	187.3	0.7	0.0194	0.0037	0.2	13.8	6.3	2.2	12.1	5.1	2.4
44	Cymoxanil	464.5	151.4	3.1	102.6	414.4	0.2	0.0032	0.0022	0.7	12.2	5.4	2.3	3.3	1.5	2.1
45	Iprodione	197.1	31.4	6.3	20.1	76.1	0.3	0.0122	0.0170	1.4	14.0	5.2	2.7	11.9	4.5	2.6
46	Linuron	2092.1	1013.4	2.1	214.1	320.9	0.7	0.0147	0.0051	0.3	14.8	6.6	2.3	17.7	8.8	2.0
47	Oxamyl	3364.3	324.9	10.4	2642.5	397.8	6.6	0.0002	0.0003	2.0	10.5	8.3	1.3	0.9	0.7	1.3
48	Propanil	1207.8	417.9	2.9	59.8	68.6	0.9	0.0076	0.0053	0.7	11.1	6.7	1.7	9.1	6.5	1.4
49	Tebuconazole	27,349.5	3044.4	9.0	1193.3	397.5	3.0	0.0017	0.0027	1.6	10.1	7.2	1.4	8.3	6.1	1.4
50	Terbuthryn	188,905.8	18,208.0	10.4	9758.9	1836.6	5.3	0.0013	0.0020	1.6	10.1	7.6	1.3	8.8	7.0	1.3
51	Thiofanate-methyl	4214.8	201.9	20.9	198.4	21.0	9.4	0.0048	0.0156	3.2	9.7	9.5	1.0	NQ	11.5	NQ
52	Kresoxim-methyl	1257.6	51.4	24.5	378.6	52.4	7.2	0.0065	0.0062	1.0	6.9	8.4	0.8	6.1	6.3	1.0
53	Carbendazim	35,312.5	2477.9	14.3	2611.3	342.4	7.6	0.0011	0.0011	1.0	10.8	8.2	1.3	4.7	3.4	1.4
54	Diazinon	135,234.5	12,429.6	10.9	5428.8	1895.9	2.9	0.0013	0.0013	1.0	9.7	8.0	1.2	8.9	7.3	1.2
55	Imidacloprid	376.1	117.1	3.2	344.8	349.6	1.0	0.0032	0.0025	0.8	9.3	6.8	1.4	3.0	2.1	1.4
56	Imazalil	12,918.6	805.3	16.0	492.9	91.0	5.4	0.0001	0.0003	2.7	1.0	0.5	1.9	0.9	0.4	2.0
57	Metsulfuron-methyl	2809.0	1177.3	2.4	441.6	755.6	0.6	0.0001	0.0009	6.7	0.8	0.8	1.1	0.3	0.3	1.0
58	Profenofos	1208.9	677.4	1.8	429.4	294.4	1.5	0.0049	0.0015	0.3	5.9	3.0	2.0	5.5	2.6	2.1
59	Propiconazole	9666.4	1219.9	7.9	212.3	39.8	5.3	0.0012	0.0023	2.0	9.4	6.7	1.4	8.4	6.1	1.4
60	Pyraclostrobine	45,027.1	9556.0	4.7	2618.3	1309.1	2.0	0.0014	0.0013	1.0	9.3	5.9	1.6	8.3	5.1	1.6
61	Triadimenol	NQ	NQ	NQ	NQ	NQ	NQ	NQ	NQ	NQ	NQ	NQ	NQ	NQ	NQ	NQ
62	Terbufos	233.8	36.1	6.5	38.0	11.1	3.4	0.0040	0.0117	2.9	7.2	6.1	1.2	6.4	5.7	1.1
63	Thiacloprid	7616.6	5542.9	1.4	845.9	1548.0	0.5	0.0013	0.0018	1.3	9.5	7.3	1.3	5.4	4.3	1.2
64	Penconazole	19,505.4	2841.9	6.9	3418.8	680.9	5.0	0.0021	0.0008	0.4	9.6	8.9	1.1	6.6	6.1	1.1
65	Pirimiphos-methyl	30,079.6	7913.1	3.8	4094.1	1251.6	3.3	0.0009	0.0025	2.9	9.5	8.5	1.1	6.9	6.1	1.1
66	Tebufenozide	3121.0	893.4	3.5	1481.0	819.3	1.8	0.0047	0.0044	1.0	10.5	10.0	1.0	7.3	7.4	1.0
67	Spirodiclofen	323.6	465.3	0.7	136.5	145.5	0.9	0.0064	0.0041	0.6	11.4	9.3	1.2	7.4	4.7	1.6
68	Cyflufenamid	2032.8	2319.3	0.9	593.5	426.0	1.4	0.0021	0.0027	1.3	10.7	8.0	1.3	11.5	8.4	1.4
69	Temephos	193.6	362.4	0.5	39.0	120.6	0.3	0.0043	0.0067	1.6	9.1	7.6	1.2	6.5	4.6	1.4
70	2,4-D	NQ	NQ	NQ	NQ	NQ	NQ	NQ	NQ	NQ	NQ	NQ	NQ	NQ	NQ	NQ
71	Chlorotoluron	12,152.1	4278.1	2.8	1384.1	791.9	1.7	0.0010	0.0021	2.2	10.1	7.9	1.3	6.4	4.9	1.3
72	Cyanazine	14,965.8	1488.5	10.1	2005.4	1362.6	1.5	0.0007	0.0022	3.1	9.3	8.0	1.2	6.0	5.1	1.2
73	Terbuthylazine	66.1	7.1	9.3	64.0	19.6	3.3	0.0128	0.0228	1.8	10.0	4.3	2.3	6.8	4.0	1.7
74	Propazine	11,310.1	3010.4	3.8	2191.0	1615.1	1.4	0.0014	0.0018	1.3	10.2	7.9	1.3	7.1	5.3	1.3
75	Atrazine	322.9	17.1	18.9	37.4	22.9	1.6	0.0002	0.0004	2.4	9.4	7.4	1.3	0.4	0.3	1.2
76	Simazine	22,136.5	1551.1	14.3	1782.3	1167.0	1.5	0.0011	0.0016	1.4	10.0	7.9	1.3	5.6	4.4	1.3
77	Isoproturon	31,525.8	7624.1	4.1	2502.9	2227.1	1.1	0.0011	0.0012	1.1	10.7	8.6	1.2	7.2	5.5	1.3
78	Fenoxycarb	4857.0	3199.8	1.5	1739.9	856.4	2.0	0.0009	0.0014	1.6	10.2	8.1	1.3	7.1	5.4	1.3
79	Epoxiconazole	24,311.3	3491.6	7.0	1503.5	267.6	5.6	0.0008	0.0009	1.1	9.7	8.7	1.1	6.8	5.8	1.2
80	Benalaxyl	14,008.5	10,303.6	1.4	1258.5	1288.6	1.0	0.0008	0.0005	0.6	9.8	8.3	1.2	7.0	5.6	1.3
81	Hexythiazox	1052.4	1766.6	0.6	702.9	1934.0	0.4	0.0014	0.0009	0.6	10.7	8.4	1.3	7.7	5.7	1.4
Minimum value		66.1	7.1	0.5	20.1	11.1	0.2	0.0001	0.0003	0.2	0.8	0.5	0.82	0.3	0.3	1.0
Maximum value		188,905.8	18,208.0	24.5	14,357.8	3745.1	11.2	0.0194	0.0228	6.7	477.4	41.4	23.38	174.1	14.2	24.8
Average value		25,191.4	3668.992	7.2	2026.4	760.1	3.3	0.0029	0.0030	1.3	28.4	7.6	3.29	20.7	5.4	3.4
*p* value		0.000001***			0.0000001***			0.359608			0.001379**			0.000170***		

*US* UniSpray ionization, *ESI* electrospray ionization, *NQ* not quantified. *, **, and ***t test is significant, highly significant, and very highly significant, respectively

### Signal-to-noise ratio

A very highly significant increase of S/N ratio of US over ESI was obtained in all the three matrices (*p* = 0.00000001 in apple and in coffee, *p* = 0.0000001 in water). The highest increase of S/N ratio was 18.3 times in apple with spinosad D, 29.4 times in coffee with spinosad D, and 11.2 times in water with fludioxonil. In average, US increased the S/N ratio more than that of ESI by 3.4-fold in apple, 3.8-fold in coffee, and 3.3-fold in water (Table 3). Lubin et al. [14] have observed similar S/N ratios between US and ESI for four out of the five prostaglandins and thromboxane compounds investigated; a distinct increase of S/N ratio with US was obtained for 11-dehydro-thromboxane B(2) (11-dTXB2).

As a result of this increase in S/N ratio with US, more compounds could be detected and quantified at low level. Table 4 presents the distribution of pesticide active ingredients which could not be recovered from pre-extraction spiked samples by using UniSpray and/or electrospray interfaces. Depending on the matrix, while imazalil, triademinol. and methomyl could only be quantified with ESI, US solely could allow the quantification of temephos, thifensulfuron, fludioxonil, bentazon, and kresoxim-methyl. A gain in the range of compounds that can be quantified just by changing the ionization source is an important benefit, especially when multiple residues have to be analyzed in single run.

**Table 4:** Distribution of the analytes not quantified in all the spiked samples with UniSpray and electrospray interfaces

Matrix	Analyte	UniSpray	Electrospray
Apple	Triademinol	✗	✗
	Temephos	✓	✗
Coffee	Thifensulfuron	✓	✗
	Fludioxonil	✓	✗
	Bentazon	✓	✗
	Thiofanate-methyl	✗	✗
	Kresoxim-methyl	✓	✗
	Imazalil	✗	✓
	Triademinol	✗	✓
	Terbufos	✗	✗
	2,4-D	✗	✗
Water	Methomyl	✗	✓
	Bentazon	✗	✗
	Triademinol	✗	✗
	2,4-D	✗	✗
Total	15	10	12

### Ion ratio

No significant difference was found between US and ESI in all the three matrices (see ESM). This can be justified by the similarities in the ionization mechanism of the two interfaces. With US, molecules of the studied pharmaceutical compounds were ionized in a similar fashion to ESI, predominantly producing protonated or deprotonated species. Adduct formation (e.g., proton and sodium adducts) and in-source fragmentation were shown to be almost identical between the two sources [6]. Additionally, the spectra generated when using US closely resemble those from ESI analyses so, although there is no voltage applied to the capillary tip, it is likely that the eluent contains ions formed from solution phase redox reactions and other physical processes. It is also possible that surface-based effects on the US impactor pin, and additional gas phase phenomena, could further contribute to ion formation [13].

### Accuracy (%recovery)

The extraction recovery percentage varied largely among the active ingredients and recovery as high as 342.9% was recorded with spinosad D in apple. Pesticides pyrimethanil, spinosad A, spinosad D, and spirodiclofen showed recoveries above 120% in most of the matrices with the two interfaces, while low recoveries were mostly obtained with metsulfuron-methyl and imazalil. As compared with ESI, recovery obtained with US showed a very highly significant increase (*p* = 0.0000002, 0.001067, and 0.000002 in apple, coffee, and water, respectively), with up to 8.8-fold increase observed in apple (spirodiclofen), up to 10.6-fold increase obtained in coffee (temephos) and up to 6.3-fold increase recorded in water (monocrotophos). However, in average, the gain in recovery percentage with US was 1.4-fold in apple, 1.9-fold in coffee, and 1.5-fold in water (see ESM). High recoveries of spinosad A and D have been previously observed [20] and may result from the ionization of spinosad from reaction with QuEChERS salts that forms a complex with a strong signal enhancement matrix effect.

### Precision (%RSD)

For the great majority of analyses, the %RSD remained below the acceptable 20% [23], except bentazon with US, and terbuthylazine, monocrotophos, terbufos, and temephos with ESI. The difference in %RSD between US and ESI was very highly significant for pesticides analyzed in apple (*p* = 0.0008) and in water (*p* = 0.0001), and was highly significant for coffee (*p* = 0.0012). In general, the two interfaces showed equal precision for pesticide residue analyses in apple, and US was 1.7 times more precise than ESI for analyses in coffee (see ESM). Lubin et al. [14] found that US offers a better precision than ESI, for three out of five prostaglandins and thromboxanes in two matrices, human plasma, and pig colon. The high values of %RSD found indicate that these pesticide chemistries favor high variations among repetitions and therefore require more refinement of the protocol for improving within-laboratory reproducibility.

### Sensitivity

For the analyses of 81 pesticides in the three matrices, lower LOQs were obtained with US; it ranged between 0.0001 and 0.0333 mg/kg, while it was between 0.0001 and 0.0478 mg/kg with ESI. However, the overall LOD and LOQ did not significantly vary between the two ionization interfaces (Table 3). For analysis of prostaglandins and thromboxanes, Lubin et al. [14] reported that sensitivity was improved for three out of five compounds measured on the UniSpray source, with an increase up to factor 5, probably due to the high signal intensity resulting in saturation phenomena. In our study, we have observed a non-significant factor 1.2 to 1.3 improvement of sensitivity with US, although a rather tremendous increase of signal intensity was obtained with this novel interface.

In fact, the gain in sensitivity with US was clear for some compounds, with improvement of LOQ as high as 8.9 times with thiofanate-methyl in apple and 6.7 times with metsulfuron-methyl in water (Table 3). This can be explained by the gain in signal intensity but this improvement could not be generalized to the total large number of analytes we screened. This clear gain in signal intensity could however result in better accuracy and precision for lower concentrations of analytes, and thus increase the sensitivity of the method. But, better sensitivity is guaranteed only if selectivity is warranted, and thus depends also on the type of mass spectrometer used (e.g., high-resolution MS, MSn, ion mobility capabilities) and the nature of the sample (background) [6]. Further investigation on a broad set of spiked concentrations is needed to draw clear conclusion on the increase in signal intensity and sensitivity observed with US in multiresidue analysis of large number of pesticides.

### Matrix effect

Matrix effect values of 100 ± 20% are considered suitable values and indicate a small ME [24]. With US, a strong signal enhancement was mostly observed in apple, the highest values were recorded with fenpropimorf in apple (634.1%), pyrimethanil in apple (616.3%), spinosad A in apple (497.3%), pyrimethanil in water (477.4%), spinosad D in apple (451.4%), and pyrimethanil in coffee (312.2%); most of the other analyses showed ME values below the lowest suitable 80% value. With ESI however, none of the value was found within the suitable range, the signal suppression was more pronounced, and the highest ME values were obtained with pyrimethanil in apple (58.8%), fenpropimorf in apple (45.4%), and pyrimethanil in water (41.4%); all the other analyses showed ME values below 30%. The difference in matrix effect between the two interfaces was highly significant in apple (*p* = 0.0020) and water (*p* = 0.0014), and very highly significant in coffee (*p* = 0.0004) (Table 3). Similar ME values were found by Chawla et al. [17] who showed that MEs were dependent on the nature of both the commodity and the analyte and observed that most of the pesticides showed signal suppression in tomato, capsicum, and cumin matrixes. They also reported very high MEs of 2360.9 and 1250.8% for quizalofop-p-tefuryl and tebuconazole, respectively.

In the case of chromatography coupled with MS, the predominant cause of ME is the presence of undesired components that co-elute in the chromatographic separation and either compete for access to the surface of the droplets and subsequent ion evaporation, or induce changes in eluent properties that are known to affect the ionization process (such as surface tension, viscosity, and volatility) [17]. For most of the analyses in our study, a high signal suppression was observed, but the ME percentages were better with US, suggesting a milder ME with the new interface. In analyzing five pesticides in six matrices, Lucini et al. [25] also observed that ME occurred as ionic suppression and was found in the range of 5 to 22% depending on the compound. For 19 pharmaceutical and biological compounds tested, a quite similar ME was observed between US and ESI, but depending on the matrix and ionization mode, a small but statistically significant lower percentage of ME could be observed for US in plasma and bile in the positive ion mode, and bile in negative ion mode [12]. The difference with our results can be due to the differences of the chemistry of the compounds tested and of the solvents we used.

### Recovery efficiency

The RE varied between 1.9 and 150.0% with US, and between 1.7 and 165.5% with ESI. Irrespective of the matrix, with US, the RE percentage of 21% of the analyses was found between the suitable RE values of 100 ± 20%, while with ESI, 24% of the analyses were suitable. A significant difference was found between the RE of US and ESI in apple (*p* = 0.023259) and water (*p* = 0.037114), while in coffee, the two interfaces showed no significant difference. But in average, no difference of RE was found between the two interfaces and in the three matrices (see ESM). Lucini et al. [25] found that REs of five pesticides in six matrices were good and substantially comparable, in the range of 93–96%. The extraction recovery measures the efficiency of the analyte extraction process during sample pre-treatment (QuEChERS extraction), and the RE measures the influence of the analyzing instrument on the recovery. This implies that the two interfaces react similarly irrespective of the analyte extraction; hence, the difference of performance will mostly be based on how the interface deals with ME.

### Process efficiency

The values of PE related to quantitative determination of pesticide residues followed the same pattern as ME. The PE was higher with US over ESI in almost all the analyses. A 3.9-fold increase was observed in apple and coffee, while the increase was 3.4-fold in water. The observed increase of PE with US was significant in apple (*p* = 0.0108), highly significant in coffee (*p* = 0.0051), and very highly significant in water (*p* = 0.0002) (Table 3). Lucini et al. [25] found more closer values (74% to 90%) of PE for analysis of five pesticides in six matrices and suggested that the differences in terms of overall PE of each compound can be ascribed to different MEs, rather than to poor recoveries due to ineffective extraction efficiencies of the QuEChERS procedure.

In our study, a tentative correlation of the evaluated performance parameters and chemical class of the active ingredients showed no correlation. Similar results were obtained by [14] who observed no correlation between signal increase and chemical structure or physicochemical data of the pharmaceutical compounds analyzed. Also, no correlation could be found between the different gains obtained with ESI or US and the molecular weight, functional groups, pKa, or logP of the studied pharmaceutical compounds. This implies that complex ionization mechanisms are involved with the UniSpray source [6].

## Conclusion

This work reports the first results of pesticide residue analysis with UniSpray, a novel API source for LC-MS, in comparison with ESI, for 81 active ingredients of diverse pesticide classes and physicochemical properties, and in three different matrices, apple, coffee, and water. The new source provided comparable and good linearity; it considerably increased the signal intensity and improved the S/N ratio. No significant effects on precision and ion ratio were found. UniSpray also offered a slight gain in the range of compounds that can be quantified, as well as in the recovery percentage. The US allowed a gain in sensitivity for many compounds, but overall, the LOD and LOQ did not significantly vary between the two ionization interfaces. Signal suppression was less pronounced with US, allowing most of the ME values to be within the acceptable range, while it was more prominent with ESI and none of the value was found within the suitable ME range. The ionization sources did not affect the RE, whereas the PE was higher with US in almost all the analyses. The studied performance parameters varied irrespectively to the chemical class of the active ingredients. For a better understanding of applications and benefits of US over ESI, further analysis of pesticides at different spiked concentrations and deep study of the ionization mechanism should be envisaged.

## Electronic supplementary material

ESM 1 (PDF 710 kb)

ESM 2 (XLSX 138 kb)

## References

[1] Hird SJ, Lau BP-Y, Schuhmacher R, Krska R (2014). Liquid chromatography-mass spectrometry for the determination of chemical contaminants in food. TrAC Trends Anal Chem.

[2] Kruve A, Kunnapas A, Herodes K, Leito I (2008). Matrix effects in pesticide multi-residue analysis by liquid chromatography-mass spectrometry. J Chromatogr A.

[3] Alder L, Greulich K, Kempe G, Vieth B (2006). Residue analysis of 500 high priority pesticides: better by GC-MS or LC-MS/MS?. Mass Spectrom Rev.

[4] Zhang K, Wong JW, Yang P, Tech K, Dibenedetto AL, Lee NS, Hayward DG, Makovi CM, Krynitsky AJ, Banerjee K, Jao L, Dasgupta S, Smoker MS, Simonds R, Schreiber A (2011). Multiresidue pesticide analysis of agricultural commodities using acetonitrile salt-out extraction, dispersive solid-phase sample clean-up, and high-performance liquid chromatography-tandem mass spectrometry. J Agric Food Chem.

[5] Stahnke H, Kittlaus S, Kempe G, Hemmerling C, Alder L (2012). The influence of electrospray ion source design on matrix effects. J Mass Spectrom.

[6] Lubin A, Bajic S, Cabooter D, Augustijns P, Cuyckens F (2017). Atmospheric pressure ionization using a high voltage target compared to electrospray ionization. J Am Soc Mass Spectrom.

[7] Thurman EM, Ferrer I, Barceló D (2001). Choosing between atmospheric pressure chemical ionization and electrospray ionization interfaces for the HPLC/MS analysis of pesticides. Anal Chem.

[8] Wang R, Zhang L, Zhang Z, Tian Y (2016). Comparison of ESI– and APCI–LC–MS/MS methods: a case study of levonorgestrel in human plasma. J Pharm Anal.

[9] Lee H, Kochhar S, Shim S (2015). Comparison of electrospray ionization and atmospheric chemical ionization coupled with the liquid chromatography-tandem mass spectrometry for the analysis of cholesteryl esters. Int J Anal Chem.

[10] Eichman HJ, Eck BJ, Lagalante AF (2017). A comparison of electrospray ionization, atmospheric pressure chemical ionization, and atmospheric pressure photoionization for the liquid chromatography/tandem mass spectrometric analysis of bisphenols. Application to bisphenols in thermal paper receipts. Rapid Commun Mass Spectrom.

[11] Garcia-Ac A, Segura PA, Viglino L, Gagnon C, Sauvé S (2011). Comparison of APPI, APCI and ESI for the LC-MS/MS analysis of bezafibrate, cyclophosphamide, enalapril, methotrexate and orlistat in municipal wastewater. J Mass Spectrom.

[12] Lubin A, De Vries R, Cabooter D, Augustijns P, Cuyckens F (2017). An atmospheric pressure ionization source using a high voltage target compared to electrospray ionization for the LC/MS analysis of pharmaceutical compounds. J Pharm Biomed Anal.

[13] Bajic S (2014). U.S. patent no. 8,809,777.

[14] Lubin A, Geerinckx S, Bajic S, Cabooter D, Augustijns P, Cuyckens F, Vreeken RJ (2016). Enhanced performance for the analysis of prostaglandins and thromboxanes by liquid chromatography-tandem mass spectrometry using a new atmospheric pressure ionization source. J Chromatogr A.

[15] Van Eeckhaut A, Lanckmans K, Sarre S, Smolders I, Michotte Y (2009). Validation of bioanalytical LC-MS/MS assays: evaluation of matrix effects. J Chromatogr B.

[16] Kebarle P, Tang L (1993). From ions in solution to ions in the gas phase: the mechanism of electrospray mass spectrometry. Anal Chem.

[17] Chawla S, Patel HK, Gor HN, Vaghela KM, Solanki PP, Shah PG (2017). Evaluation of matrix effects in multiresidue analysis of pesticide residues in vegetables and spices by LC-MS/MS. J AOAC Int.

[18] King R, Bonfiglio R, Fernandez-Metzler C, Miller-Stein C, Olah T (2000). Mechanistic investigation of ionization suppression in electrospray ionization. J Am Soc Mass Spectrom.

[19] Kouzayha A, Rahman Rabaa A, Al Iskandarani M, Beh D, Budzinski H, Jaber F (2012). Multiresidue method for determination of 67 pesticides in water samples using solid-phase extraction with centrifugation and gas chromatography-mass spectrometry. Am J Anal Chem.

[20] Galani JHY, Houbraken M, Wumbei A, Djeugap FJ, Fotio D, Spanoghe P (2018). Evaluation of 99 pesticide residues in major agricultural products from the Western Highlands Zone of Cameroon using QuEChERS method extraction and LC-MS/MS and GC-ECD analyses. Foods.

[21] Corley J (2003). Best practices in establishing detection and quantification limits for pesticide residues in foods. Handb Residue Anal Methods Agrochem.

[22] Matuszewski BK, Constanzer ML, Chavez-Eng CM (2003). Strategies for the assessment of matrix effect in quantitative bioanalytical methods based on HPLC-MS/MS. Anal Chem.

[23] European Commission (2015) Guidance document on analytical quality control and method validation procedures for pesticides residues analysis in food and feed.

[24] Cuadros-Rodríguez L, García-Campaña AM, Almansa-López E, Egea-González FJ, Castro Cano ML, Garrido Frenich A, Martínez-Vidal JL (2003). Correction function on biased results due to matrix effects: application to the routine analysis of pesticide residues. Anal Chim Acta.

[25] Lucini L, Pietro MG (2011). Performance and matrix effect observed in QuEChERS extraction and tandem mass spectrometry analyses of pesticide residues in different target crops. J Chromatogr Sci.

