# Supplementary material for: Comparison of electrospray and UniSpray, a novel atmospheric pressure ionization interface, for LC-MS/MS analysis of 81 pesticide residues in food and water matrices
Source: Anal Bioanal Chem. 2019 May 31;411(20):5099–113. doi: 10.1007/s00216-019-01886-z (PMC6647134; doi:10.1007/s00216-019-01886-z)
Supplement: Supplementary file 1 — (PDF 710 kb) [file 216_2019_1886_MOESM1_ESM.pdf]

Analytical and Bioanalytical Chemistry

Electronic Supplementary Material

Comparison of electrospray and UniSpray, a novel atmospheric pressure ionization interface, for LC-MS/MS analysis of 81 pesticide residues in food and water matrices

Joseph Hubert Yamdeu Galani, Michael Houbraken, Marijn Van Hulle, Pieter Spanoghe

Additional file available under 10.1007/s00216-019-01886-z.
